# Increased Serum Lipopolysaccharide Levels are Related to a Higher Prevalent Risk of Subclinical Hypothyroidism

**DOI:** 10.1155/ije/6332634

**Published:** 2025-10-22

**Authors:** Xuan An, Xiaoyi Wang, Jin Zhang, Mingtong Xu, Muchao Wu

**Affiliations:** Department of Endocrinology, Sun Yat-Sen Memorial Hospital, Sun Yat-Sen University, Guangzhou 510120, China

**Keywords:** hashimoto's thyroiditis, lipopolysaccharide, subclinical hypothyroidism, thyroid autoantibody

## Abstract

**Background:**

This study aimed to assess the relationship between serum lipopolysaccharide levels and subclinical hypothyroidism in a southern Chinese adult population.

**Methods:**

This cross-sectional community-based study included 2577 participants. Fasting venous samples were taken to examine lipopolysaccharide, thyroid hormone, thyroglobulin antibody, and thyroid peroxidase antibody levels. The population was divided into quartiles according to serum lipopolysaccharide levels. A multivariable-adjusted logistic regression model was applied to test the association between lipopolysaccharide and subclinical hypothyroidism.

**Results:**

Participants with increased serum levels of lipopolysaccharide had a higher prevalence of Hashimoto's thyroiditis (Q1: 10.4%, Q2: 14.3%, Q3: 17.8%, and Q4: 29.5%; *p*-trend < 0.001) and subclinical hypothyroidism (Q1: 2.2%, Q2: 4.7%, Q3: 7.5%, and Q4: 13.4%; *p-*trend < 0.001). The multivariable-adjusted odds ratios for subclinical hypothyroidism in the 2nd, 3rd, and 4th serum lipopolysaccharide quartiles were 1.979 (95% CI: 1.033–3.793), 2.867 (95% CI: 1.534–5.360), and 4.091 (95% CI: 2.198–7.613), *p-*trend < 0.001, respectively, compared to the 1st quartile.

**Conclusions:**

Increased serum lipopolysaccharide levels were related to an increased prevalence of subclinical hypothyroidism in the southern Chinese adult population.

## 1. Introduction

Hypothyroidism is classified into subclinical (SCH) and clinical (or overt) hypothyroidism based on the blood levels of thyroid hormones. SCH, defined as elevated serum thyroid–stimulating hormone (TSH) concentrations accompanied by normal free thyroxine (FT4) concentrations, is more common than clinical hypothyroidism, affecting up to 10% of adults [1]. SCH is related to a higher risk of heart diseases and related mortality. Similar to clinical hypothyroidism, SCH most often results from Hashimoto's thyroiditis (HT) [1].

HT and Graves' disease, which are the primary causes of hyperthyroidism, are the two most common autoimmune thyroid diseases (AITDs) [2]. Apart from genetic susceptibility, environmental factors, such as radiation, medication, viral infection, iodine, and selenium, contribute to the development of AITDs [3]. In recent years, some studies have revealed that gut microbiota and metabolites are another potential environmental risk factor for AITDs [3–5]. The association between gut microbiota and HT is well-documented [5, 6], and intestinal dysbiosis is also associated with clinical hypothyroidism or SCH [7–11].

However, the mechanisms underlying the relationship between intestinal dysbiosis and AITDs remain poorly understood. One of the possible mechanisms is that gut dysbiosis and intestinal barrier dysfunction in patients with AITD lead to the translocation of lipopolysaccharide (LPS), the principal component of gut Gram-negative bacteria, into the systemic circulation. LPS may alter the balance of immune-regulatory T or B lymphocyte subsets, promoting the production of autoantigens and cytokines in the thyroid [7, 9, 11–15].

Patients with hypothyroidism have been observed to have increased blood LPS levels [7]. LPS may contribute to the pathogenesis of hypothyroidism. For example, 48 h of LPS infusion in vivo decreases blood FT4 levels in pigs [16]. In addition, HT is the main cause of hypothyroidism, and experimental autoimmune thyroiditis in mice, a common animal model of HT, has been established via injection of LPS and mouse thyroglobulin [17]. However, population-based evidence linking serum LPS levels with SCH remains limited, especially in Chinese cohorts.

Therefore, in this study, we aimed to evaluate the relationship between serum LPS levels and SCH in a community-based population in Guangzhou, southern China.

## 2. Materials and Methods

### 2.1. Study Population

This was a cross-sectional study, which was based on a 2015 epidemiological survey on thyroid disorders in Guangzhou. Of the 2720 residents aged over 18 years who participated, exclusions were made for missing general information (*n* = 5); lack of information on previous thyroid diseases (*n* = 10), self-reported hyperthyroidism, hypothyroidism, or thyroid carcinoma, who might take antihyperthyroidism drugs or thyroid hormones (*n* = 90); and missing values of urinary iodine concentrations (UICs), TSH, or high-sensitivity C-reactive protein (hs-CRP) (*n* = 11), or hs-CRP ≥ 10 mg/L (*n* = 27, indicating acute and active infection), leaving 2577 participants for the final analyses. Informed consent forms were signed by all study participants. The protocol of the study was approved by the Medical Ethics Committee of Sun Yat-Sen Memorial Hospital (No. 2014, [33]).

### 2.2. Data Collection and Sample Measurements

Information regarding sex, age, drinking (yes or no), smoking (yes or no), and history of thyroid disease was collected using a questionnaire. Anthropometric measurements of height and weight were performed in all participants, and body mass index (BMI) was calculated. Morning spot urine samples were obtained. Venous samples were drawn after overnight fasting for 8–10 h. Then, an oral glucose tolerance test (OGTT) was performed, and a blood sample was withdrawn 120 min postglucose administration. UIC, serum levels of FT4, free triiodothyronine (FT3), TSH, thyroglobulin antibodies (TG-Ab), thyroid peroxidase antibodies (TPO-Ab), lipid, alanine aminotransferase (ALT), creatinine (Cr), and plasma glucose level were tested as described previously [18, 19]. Serum TSH, TG-Ab, and TPO-Ab levels were measured in all participants. If the TSH level was more than 4.20 mU/L, FT4 was measured, while if the TSH was less than 0.27 mU/L, then both FT3 and FT4 were tested in the same sample. Serum LPS and hs-CRP levels were assessed as described in our previous study [20]. In brief, serum LPS was measured using the Limulus Amebocyte Lysate assay (Hycult Biotech, Uden, the Netherlands), and serum hs-CRP was tested using the latex-enhanced immunoturbidimetric assay (Ningbo Medical System Biotechnology Co., Ltd., Zhejiang, China).

### 2.3. Definition of SCH, HT, Diabetes, and Dyslipidemia

SCH was diagnosed if serum TSH levels were > 4.20 mU/L with normal serum FT4 level (10.3–24.5  pmol/L) [18]. Serum level of TG-Ab ≥ 50 IU/mL, TPO-Ab ≥ 34 IU/mL, or both was diagnosed as HT [18, 21, 22]. The following criteria were used for diagnosing diabetes: fasting plasma glucose ≥ 7.0 mmol/L, OGTT 2 h-plasma glucose ≥ 11.1 mmol/L, or self-reported diabetes history. Dyslipidemia was defined as total cholesterol > 5.17 mmol/L, triglyceride > 1.70 mmol/L, low-density lipoprotein cholesterol > 3.37 mmol/L, high-density lipoprotein cholesterol < 1.04 mmol/L, or if participants were taking lipid-lowering drugs [19].

### 2.4. Statistical Analysis

Continuous variables are presented as mean ± SD or median (interquartile range), while categorical variables are presented as a proportion (%). The population was divided into quartiles according to serum LPS levels (Q1: < 0.26, Q2: 0.26–0.36, Q3: 0.37–0.65, and Q4: > 0.65 EU/mL). Because the variables of hs-CRP, UIC, FT4, and TSH were non-normally distributed, a logarithmic transformation was performed, and then *p* for the trends across the groups was calculated by linear regression analysis; while the Mantel–Haenszel chi-square test was used for categorical variables. A univariable binary logistic regression model was applied to identify potential risk factors for SCH. Unadjusted and multivariable-adjusted binary logistic regression models were applied to assess SCH prevalence. In addition, the population was also classified by age (< 40 and ≥ 40 years), sex (female and male), and BMI (< 25 and ≥ 25 kg/m^2^), and then further stratified by the quartile levels of LPS in each subgroup; multivariable-adjusted binary logistic regression models were applied to calculate the odds ratios (ORs) for SCH and *p* for the trends in each subgroup. All tests were two-sided, and the level of significance was set at *p* < 0.05.

## 3. Results

### 3.1. Clinical and Biochemical Characteristics of the Study Population

The study population had a mean age of 45.71 ± 15.68 years, and 43.1% were male. The prevalence of SCH was 6.9%.  Table 1 displays the clinical and biochemical features of the total study population according to the quartile levels of serum LPS. As serum LPS levels increased, the following trends were observed: hs-CRP levels rose (Q1: 0.54 [0.30–0.96] mg/L, Q2: 0.87 [0.47–1.21] mg/L, Q3: 1.33 [0.96–2.55] mg/L, and Q4: 2.11 [1.13–3.77] mg/L; *p*-trend < 0.001) and the prevalence of positive TG-Ab (Q1: 7.3%, Q2: 11.3%, Q3: 13.5%, and Q4: 25.9%; *p*-trend < 0.001), positive TPO-Ab (Q1: 5.6%, Q2: 7.8%, Q3: 11.2%, and Q4: 20.3%; *p*-trend < 0.001), and HT (Q1: 10.4%, Q2: 14.3%, Q3: 17.8%, and Q4: 29.5%; *p*-trend < 0.001) increased. Similarly, serum TSH levels (Q1: 1.75 (1.26–2.42) mU/L, Q2: 1.78 [1.25–2.56] mU/L, Q3: 1.88 [1.23–2.65] mU/L, and Q4: 2.06 [1.30–3.20] mU/L; *p*-trend < 0.001) and the prevalence of SCH (Q1: 2.2%, Q2: 4.7%, Q3: 7.5%, and Q4: 13.4%; *p*-trend < 0.001) increased, while FT4 levels decreased (Q1: 16.96 [14.16–19.27] pmol/L, Q2: 16.26 [13.83–18.21] pmol/L, Q3: 16.23 [13.43–21.52] pmol/L, and Q4: 15.80 [13.88–17.93] pmol/L; *p-*trend = 0.010).

### 3.2. Risk Factors for SCH by Logistic Regression Analysis

The following significant risk factors for SCH were identified by univariable logistic regression analysis: sex (OR: 1.619, 95% CI: 1.171–2.237, *p*=0.004), smoking (OR: 4.667, 95% CI: 2.279–9.556, *p* < 0.001), dyslipidemia (OR: 1.529, 95% CI: 1.076–2.174, *p*=0.018), hs-CRP (OR: 1.242, 95% CI: 1.151–1.340, *p* < 0.001), UIC (OR: 1.001, 95% CI: 1.000–1.001, *p*=0.009), positive TG-Ab (OR: 3.280, 95% CI: 2.349–4.580, *p* < 0.001), positive TPO-Ab (OR: 3.092, 95% CI: 2.155–4.435, *p* < 0.001), HT (OR: 2.730, 95% CI: 1.971–3.779, *p* < 0.001), and serum LPS (OR: 2.713, 95% CI: 2.123–3.468, *p* < 0.001) (Table 2).

### 3.3. The Relationship Between Serum LPS and SCH

**Figure 1:**
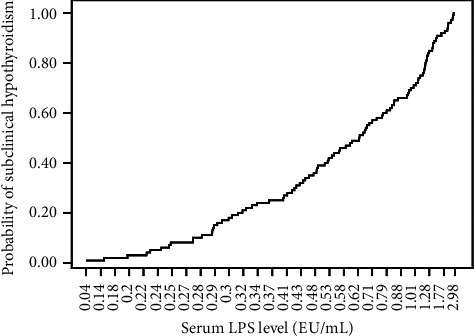
Probability of subclinical hypothyroidism and serum levels of LPS in 2577 Chinese adults. LPS: lipopolysaccharide.

We assessed the relationship between serum LPS and SCH. As presented in Figure 1, increased serum LPS levels were related to a higher risk of SCH.

**Figure 2:**
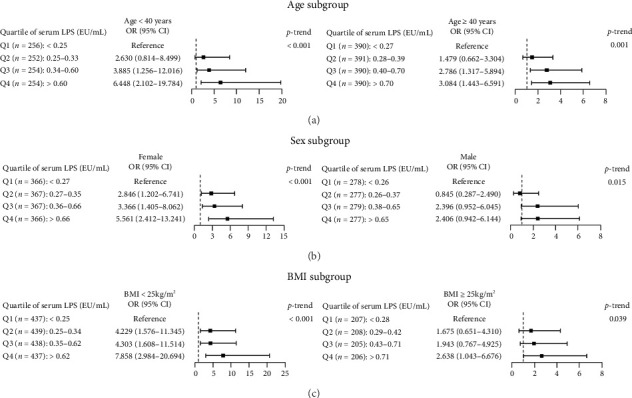
Odds ratios (ORs) for subclinical hypothyroidism according to LPS in the age, sex, and BMI subgroups. The population was also classified by age, sex, and BMI, and then further stratified by the quartile levels of LPS in each subgroup. *p* for the trend in each subgroup was calculated by the binary logistic regression model. The ORs were adjusted for age, or gender, or BMI, and alcohol consumption, smoking status, alanine aminotransferase, creatinine, diabetes, dyslipidemia, high-sensitivity C-reactive protein, urinary iodine concentration, positive thyroglobulin antibody, and positive thyroid peroxidase antibody. (a) Age subgroup (< 40 and ≥ 40 years), (b) Sex subgroup (female and male). (c) BMI subgroup (< 25 and ≥ 25 kg/m^2^). LPS: lipopolysaccharide; BMI: body mass index.

A logistic regression model was applied to further evaluate the relationship between serum LPS levels and SCH. In the unadjusted model, the OR for SCH in participants in the 2nd, 3rd, and 4th quartiles of serum LPS were 2.202 (95% CI: 1.157–4.193), 3.630 (95% CI: 1.981–6.652), and 6.946 (95% CI: 3.904–12.360), *p-*trend < 0.001, respectively. After adjusting for the effects of age, gender, BMI, alcohol consumption, smoking status, ALT, Cr, diabetes, dyslipidemia, hs-CRP, UIC, positive TG-Ab, and positive TPO-Ab, the ORs for SCH in the 2nd, 3rd, and 4th quartile levels of serum LPS were 1.979 (95% CI: 1.033–3.793), 2.867 (95% CI: 1.534–5.360), and 4.091 (95% CI: 2.198–7.613), *p-*trend < 0.001, respectively, compared to the 1st quartile levels of serum LPS (Table 3). The increasing trend of multivariable-adjusted ORs for SCH with increased quartile of LPS levels was also observed in the subgroups of age (< 40 or ≥ 40 years), sex (female or male), and BMI (< 25 or ≥ 25 kg/m^2^) (all *p*-trend < 0.05) (Figure 2).

## 4. Discussion

In the current study, we reported that increased serum LPS levels were related to an increased prevalence of positive TG-Ab, positive TPO-Ab, and HT. Meanwhile, increased serum LPS levels were also associated with increased serum TSH, decreased serum FT4 levels, and an increased prevalence of SCH. After adjustment, the risk in participants with the highest quartile of serum LPS was 4.091 times higher than in those with the lowest quartile. These findings indicate that increased serum LPS levels are related to a higher prevalence of SCH in Chinese adults.

SCH is characterized by mild thyroid dysfunction; however, it may increase the incidence of heart disease and related mortality [1]. In addition, it is associated with cognitive impairment and cerebral venous thrombosis [23, 24]. During pregnancy, SCH is a risk factor of spontaneous abortion, fetal growth restriction, prematurity, low birth weight, and neonatal death [25].

Approximately 2%–5% of SCH cases progress to clinical hypothyroidism annually [26]. The causes of SCH and clinical hypothyroidism vary, while HT is considered the main cause [1]. The mechanisms underlying HT and autoimmune hypothyroidism remain unclear. Accumulating evidence indicates that human intestinal microbiota is associated with HT and autoimmune hypothyroidism [5, 7]. The gut microbiome may affect thyroid function via the “gut–thyroid axis” [5, 7]. Recently, Su et al. [7] reported that gut dysbiosis was associated with the clinical features of hypothyroidism, and transplantation of intestinal flora from patients with hypothyroidism might decrease thyroid function in mice. Li et al. [9] observed that differential metabolites in gut microflora are related to maternal outcomes in SCH during pregnancy.

Short-chain fatty acids (SCFAs) are gut bacterial metabolites, and reduced SCFA production is associated with decreased intestinal barrier [5]. Intestinal bacteria that produce SCFA are reduced in patients with hypothyroidism [7]. Some researchers have speculated that a decrease in the intestinal barrier might lead to an influx of intestinal LPS into the systemic circulation [5, 7]. Patients with clinical hypothyroidism have higher blood LPS levels than healthy individuals, and mice with intestinal microbiota transplanted from patients with clinical hypothyroidism have higher blood LPS levels than mice with intestinal microbiota transplanted from healthy individuals [7]. In this study, increased blood LPS levels were related to an increased risk of SCH. Collectively, these results indicate that LPS plays an important role in hypothyroidism, including SCH.

In addition to being an indicator of translocation of gut bacteria and their metabolites, LPS is a potent inducer of inflammation. In vitro, LPS increases the expression of transforming growth factor-β1 (TGF-β1) in peripheral blood mononuclear cells from patients with HT, which might contribute to differentiation from naive T cells into Th17 cells [12]. The LPS receptor, Toll-like receptor 4, is expressed in thyroid follicular cells, and LPS increases TPO (a major thyroid autoantigen) expression via the NF-κB pathway [14]. In addition, LPS increases the expression of tumor necrosis factor-α (TNF-α) in rat thyroid cells, while serum TNF-α levels are associated with TG-Ab levels in patients with HT, and serum TNF-α levels are also increased in patients with SCH due to autoimmune thyroiditis [27–29]. In this study, we found that increased blood LPS levels were related to an increased prevalence of HT and SCH, indicating a potential association between LPS, HT, and SCH. LPS might cause inflammation of the thyroid and then lead to the development of SCH.

Graves' hyperthyroidism and autoimmune hypothyroidism represent opposing thyroid functional states. Graves' hyperthyroidism is characterized by elevated FT3 and FT4 levels and decreased TSH levels, whereas autoimmune hypothyroidism is marked by decreased blood FT4 and elevated TSH levels. However, both conditions are associated with increased blood LPS levels [7, 30]. In addition, a recent study by Fuke et al. [31] reported a positive association between blood LPS-binding protein (LBP) (a plasma protein mediating immune responses triggered by LPS) and FT4 (but not TSH) levels in a Japanese adult population, suggesting that LPS may have an effect on the development of Graves' hyperthyroidism. Conversely, the present study showed that increased serum LPS levels were related to decreased FT4 and increased TSH levels in a Chinese population, indicating that LPS may affect the development of autoimmune hypothyroidism. The cause of these inconsistent findings remains unknown, and the effects of LPS on the development of Graves' hyperthyroidism and autoimmune hypothyroidism require further exploration.

This study has some limitations. First, it was conducted in an iodine-sufficient area in Southern China, which may limit the generalizability of these findings. Second, some factors (such as using antibiotics, probiotics, or immunomodulatory drugs; or chronic inflammatory diseases, etc.) that could affect serum LPS levels were not considered. Third, some inflammatory mediators (such as interleukin-6 and TNF-α, which would further help to elucidate the mechanisms of LPS in the development of SCH) were not detected, and FT4 levels were only measured in participants with elevated TSH levels. Finally, as a cross-sectional study, the causality between LPS and SCH could not be established. Future research is necessary to identify the effects of LPS on the development of SCH.

## 5. Conclusion

The current study results suggest that increased serum LPS levels were related to an increased prevalence of HT and SCH in Chinese adults. Further prospective and pathophysiological studies are required to verify these findings and illustrate the potential mechanisms.

## Figures and Tables

**Table 1:** Clinical and biochemical characteristics of the study population by serum LPS quartiles (EU/mL).

Characteristics	Q1 < 0.26 (*n* = 645)	Q2 0.26–0.36 (*n* = 644)	Q3 0.37–0.65 (*n* = 644)	Q4 > 0.65 (*n* = 644)	*p*-trend
Age (years)	43.53 ± 15.13	44.57 ± 15.71	46.15 ± 15.51	48.60 ± 15.94	< 0.001
Female, *n* (%)	357 (55.3)	387 (60.1)	351 (54.5)	371 (57.6)	0.891
BMI (kg/m^2^)	22.98 ± 3.40	23.11 ± 3.66	23.96 ± 3.44	24.07 ± 3.91	< 0.001
Drinking, *n* (%)	370 (57.4)	310 (48.1)	303 (47.0)	299 (446.4)	< 0.001
Smoking, *n* (%)	125 (19.4)	104 (16.1)	113 (17.5)	98 (15.2)	0.094
ALT (U/L)	19.54 ± 12.90	20.36 ± 16.68	21.73 ± 17.85	22.71 ± 17.85	< 0.001
Cr (μmol/L)	95.26 ± 23.09	93.75 ± 19.57	94.57 ± 22.25	95.12 ± 20.97	0.913
Diabetes, *n* (%)	48 (7.4)	54 (8.4)	73 (11.3)	107 (16.6)	< 0.001
Dyslipidemia, *n* (%)	376 (58.3)	400 (62.1)	454 (70.5)	489 (75.9)	< 0.001
hs-CRP (mg/L)	0.54 (0.30–0.96)	0.87 (0.47–1.21)	1.33 (0.96–2.55)	2.11 (1.13–3.77)	< 0.001
UIC (μg/L)	124.0 (84.7–176.5)	130.0 (90.3–192.0)	130.0 (88.1–175.9)	130.0 (87.0–185.8)	0.084
Positive TG-Ab, *n* (%)	47 (7.3)	73 (11.3)	87 (13.5)	167 (25.9)	< 0.001
Positive TPO-Ab, *n* (%)	36 (5.6)	50 (7.8)	72 (11.2)	131 (20.3)	< 0.001
HT (*n* (%))	67 (10.4)	92 (14.3)	115 (17.8)	190 (29.5)	< 0.001
FT4 (pmol/L)^#^	16.96 (14.16–19.27)	16.26 (13.83–18.21)	16.23 (13.43–21.52)	15.80 (13.88–17.93)	0.010
TSH (mU/L)	1.75 (1.26–2.42)	1.78 (1.25–2.56)	1.88 (1.23–2.65)	2.06 (1.30–3.20)	< 0.001
SCH, *n* (%)	14 (2.2)	30 (4.7)	48 (7.5)	86 (13.4)	< 0.001

*Note:p* for the trend across the groups was calculated by the linear regression analysis (for the normally distributed continuous variables, or after logarithmic transformation for the non-normally distributed continuous variables) or the Mantel–Haenszel chi-square method (for the categorical variables). LPS, lipopolysaccharide; ALT, alanine aminotransferase; Cr, creatinine; TG-Ab, thyroglobulin antibody; TPO-Ab, thyroid peroxidase antibody; FT4, free thyroxine; SCH, subclinical hypothyroidism.

Abbreviations: BMI, body mass index; hs-CRP, high-sensitivity C-reactive protein; HT, Hashimoto's thyroiditis; TSH, thyroid-stimulating hormone; UIC, urinary iodine concentration.

^*#*^FT4 was measured only in participants with elevated TSH levels, leaving 224 in the analyses.

**Table 2:** Risk factors of subclinical hypothyroidism by univariable logistic regression analysis.

Characteristics	*B*	SE	OR	95% CI	*p* value
Age^∗^	0.004	0.159	1.005	0.736–1.372	0.977
Sex^∗^	0.482	0.165	1.619	1.171–2.237	0.004
BMI^∗^	0.213	0.162	1.237	0.901–1.699	0.188
Drinking^∗^	0.264	0.157	1.303	0.959–1.770	0.091
Smoking^∗^	1.541	0.366	4.667	2.279–9.556	< 0.001
ALT	−0.008	0.006	0.992	0.981–1.004	0.190
Cr	0.002	0.004	1.002	0.995–1.009	0.556
Diabetes^∗^	0.359	0.222	1.432	0.926–2.214	0.106
Dyslipidemia^∗^	0.425	0.179	1.529	1.076–2.174	0.018
hs-CRP	0.216	0.039	1.242	1.151–1.340	< 0.001
UIC	0.001	0.000	1.001	1.000–1.001	0.009
Positive TG-Ab^∗^	1.188	0.170	3.280	2.349–4.580	< 0.001
Positive TPO-Ab^∗^	1.129	0.184	3.092	2.155–4.435	< 0.001
HT^∗^	1.004	0.166	2.730	1.971–3.779	< 0.001
LPS	0.998	0.125	2.713	2.123–3.468	< 0.001

*Note:* ALT, alanine aminotransferase; Cr, creatinine; LPS, lipopolysaccharide; TG-Ab, thyroglobulin antibody; TPO-Ab, thyroid peroxidase antibody.

Abbreviations: BMI, body mass index; hs-CRP, high-sensitivity C-reactive protein; HT, Hashimoto's thyroiditis; UIC, urinary iodine concentration.

^∗^Processing as classification variables: age (≥ 40 and < 40 years), sex (male and female), BMI (≥ 25 and < 25 kg/m^2^), drinking status (drinking and nondrinking), and smoking status (smoking and nonsmoking).

**Table 3:** Association of serum LPS levels with subclinical hypothyroidism.

Quartile of serum LPS (EU/mL)	Unadjusted model OR (95% CI)	Adjusted model^∗^
Q1 (*n* = 645): < 0.26	1	1
Q2 (*n* = 644): 0.26–0.36	2.202 (1.157–4.193)	1.979 (1.033–3.793)
Q3 (*n* = 644): 0.37–0.65	3.630 (1.981–6.652)	2.867 (1.534–5.360)
Q4 (*n* = 644): > 0.65	6.946 (3.904–12.360)	4.091 (2.198–7.613)
*p*-trend	< 0.001	< 0.001

*Note:p* for the trend across the groups was calculated by the binary logistic regression model. LPS, lipopolysaccharide.

^∗^Adjusted for age, gender, body mass index, alcohol consumption, smoking status, alanine aminotransferase, creatinine, diabetes, dyslipidemia, high-sensitivity C-reactive protein, urinary iodine concentration, positive thyroglobulin antibody, and positive thyroid peroxidase antibody.

## Data Availability

The data used to support the study are available from the corresponding author upon request.

## Nomenclature

SCH: Subclinical hypothyroidism
TSH: Thyroid-stimulating hormone
FT4: Free thyroxine
HT: Hashimoto's thyroiditis
AITDs: Autoimmune thyroid diseases
LPS: Lipopolysaccharide
UIC: Urinary iodine concentration
hs-CRP: High-sensitivity C-reactive protein
OGTT: Oral glucose tolerance test
BMI: Body mass index
FT3: Free triiodothyronine
TG-Ab: Thyroglobulin antibody
TPO-Ab: Thyroid peroxidase antibody
SCFAs: Short-chain fatty acids
LBP: Lipopolysaccharide binding protein
TGF-β1: Transforming growth factor-β1
TNF-α: Tumor necrosis factor-α
